# Host-parasite association in trombiculid mites (Actinotrichida: Trombiculidae) of temperate zone - the case of *Hirsutiella zachvatkini* (Schluger, 1948); are we dealing with prolonged contact with the host?

**DOI:** 10.1186/s13071-016-1339-2

**Published:** 2016-02-02

**Authors:** Hanna Moniuszko, Joanna Mąkol

**Affiliations:** Department of Invertebrate Systematics and Ecology, Institute of Biology, Wrocław University of Environmental and Life Sciences, Kożuchowska 5b, 51-631 Wrocław, Poland

**Keywords:** Chiggers, Parasitic phase, Prevalence, Intensity of infection, Abundance, Topic preferences, Rodents

## Abstract

**Background:**

The time-extended contact of trombiculid larvae with hosts poses a question of its ecological determinants. The phenomenon, which may facilitate the overwintering of larvae in the temperate zone, was previously observed in few parasitengone taxa, but not confirmed for mammal-associated trombiculids. The study aims at tracing the phenology of larvae of *Hirsutiella zachvatkini* and at verifying the hypothesis of contact with the host, extending beyond the parasitic phase.

**Methods:**

*Apodemus agrarius*, *Apodemus flavicollis* and *Myodes glareolus*, trapped during 2-year studies, were checked for the presence of trombiculid larvae. Larvae of *H. zachvatkini* served for the studies. The degree of mites’ engorgement was checked over time in order to estimate the duration of feeding phase and to measure the maximum size increase. The experimental rearing aimed at ascertaining the relations between the level of engorgement and successful transformation of larva into subsequent instar.

**Results:**

The mass appearance of larvae on hosts fell on autumn and winter, with a decrease observed in spring, leading to an almost total absence in early and mid summer. The highest intensity, attained in late autumn or in winter, was not followed by further increase in the number of host-associated larvae. The percentage of unengorged larvae on hosts was disproportionately small, irrespective of the season. The size increase of larva was 12.6-fold at maximum. Engorged or partly engorged larvae, observed from the beginning of mass appearance over the entire period of host-parasite association in the field, transformed into subsequent instar when removed from host.

**Conclusions:**

An increase in intensity observed from the onset of appearance of larvae on hosts, through autumn and winter months, at rarity of observations of unengorged larvae and absence of engorged larvae off-host, indicates a prolonged contact with hosts, aimed at synchronisation of life cycle, conditioned by food resources available for active postlarval forms and constitutes a strategy enabling larvae to survive the unfavourable winter conditions. The proportion of engorged and partly engorged vs. unfed larvae, observed over the survey, along with their ability to transform into subsequent instars, indicates a relatively short feeding phase. The lack of continuous increase in abundance and intensity towards spring and summer suggests a gradual detachment of partly and fully engorged larvae which attained the readiness to subsequent development. The size increase of larvae during their parasitic phase does not corroborate the neosomy in *H. zachvatkini*. Host-associated differences in topic preferences of the chiggers become less obvious at maximum infection rates. Quantitative descriptors of parasite population place *M. glareolus* among the most infected hosts of *H. zachvatkini* in contrast to *Apodemus* mice collected in the same habitat.

## Background

Trombiculid mites (Actinotrichida: Parasitengona, Trombiculidae), with more than 3000 species distributed worldwide, are characterized by a complex life cycle comprising egg, prelarva, larva, three nymphal instars and adult, the larva, deutonymph and adult being the only active instars and the larva being the only parasitic and, with few exceptions, vertebrate-associated instar. During the parasitic phase the contact of larva with the host is facilitated by the formation of stylostome, a tube extending between the larva’s mouthparts and the hosts’ dermis which, besides food intake, ensures firm attachment to the host’s tissues. A comprehensive survey of stylostome formed by trombiculid mites was provided by Shatrov [1].

Earlier observations made it possible to ascertain the approximate time of effective feeding of trombiculid larvae which, according to different authors, is 3–5 days [2] 5–12 days [3] or, in the case of *Leptotrombidium* spp., 2–7 days [4]. Shatrov [5] reported 4 days of effective feeding for *Hirsutiella zachvatkini* parasitizing guinea pig. The mean feeding time reported by Harrison [6, 7] for 16 trombiculid species varied between 2.1 and 20 days, with the maximum value attained at 60 days. Several reports, however, suggest a possibility of prolonged association of parasites with their hosts, extending beyond the actual parasitic phase. According to Wohltmann [8], the body mass and morphological constitution of vertebrates created new evolutionary possibilities for parasites and induced evolutionary changes, among which the prolonged host-parasite association could be listed.

Williams [9] observed larvae of *Eutrombicula alfreddugesi* (Oudemans, 1910) overwintering deep in the ears of their mammalian hosts, whereas Takahashi et al. [10] reported the time-extended host-parasite association for *Leptotrombidium pallidum* (Nagayo, Mitamura et Tamiya, 1919). This phenomenon was recorded also for trombiculids associated with amphibians, reptiles and birds; for example, larvae of *Hannemania hegeneri* (Hyland, 1956), after artificially induced infection, remained on *Rana* spp. for up to six months, which was much longer than the time required for effective feeding [11]. Another anuran parasite, *Endotrombicula pillersi* (Sambon, 1928), was reported to remain on its host for up to three years [12]. As far as lizard hosts are concerned, larvae of *Eutrombicula lipovskyana* (Wolfenbarger, 1953) remained attached to *Sceloporus jarrovii* for 52 days [13], whereas those of *Neotrombicula harperi* (Ewing, 1928) were associated with *Uta stansburiana* for 30–90 days [14]. Literak et al. [15] recorded the occurrence of *Ascoschoengastia latyshevi* (Schluger, 1955) on hole-nesting birds from December to March, however neither here nor in the preceding cases was there strict evidence of associations encompassing both the actual feeding and one extending beyond the parasitic phase. The prolonged host-parasite contact was also reported for a few representatives of parasitengone families with invertebrate-associated larvae, for example, terrestrial larvae of *Neothrombium neglectum* (Bruyant, 1909) (Neothrombiidae) were reported to remain on a mole cricket for up to 198 days before reaching the subsequent instar [16], whereas water mite larvae of *Eylais* sp. (Eylaidae) started their actual feeding in spring, after hibernation on their corixid hosts [8]. The experiments carried out by Wohltmann [17] on *Eutrombidium trig*onum revealed, however, that unfed, not host-associated larvae were unlikely to hibernate.

According to Wohltmann [8], the prolonged host-parasite association, still insufficiently studied as far as biology of many parasitengone taxa is concerned, ensures suitable environment for the larvae, enabling them to survive winter in the temperate zone. The ecological background of the strategy, however, may vary depending on the host group. The larva, being especially sensitive to adverse environmental factors, seems to encounter the most favourable thermal conditions during winter both on the bodies of mammalian hosts and within their burrows. The latter coincides with the rarity of records of free, non-host-associated parasitengone larvae as hibernating in the temperate zone. A review of the hitherto records was provided by Wohltmann [8] and Belozerov [18].

The present work results from two-year monitoring of rodent-associated trombiculid larvae, parasites of the striped field mouse, *Apodemus agrarius* (Pallas, 1771)*,* yellow-necked mouse, *A. flavicollis* (Melchior, 1834)*,* and bank vole, *Myodes glareolus* (Schreber, 1780). The study aims at verification of the hypothesis on the prolonged contact with the host, extending beyond the parasitic phase and not verified for mammal-associated trombiculids since the reports of Williams [9] and Takahashi et al. [10]. Factors influencing the site preferences of larvae as well as the prevalence and intensity of infection are considered.

## Methods

The research was carried out from September 2012 to September 2014, with catches scheduled at 2–4 weeks intervals. The material comprised 32 specimens of *A. agrarius*, 31 of *A. flavicollis* and 56 of *M. glareolus* examined for the presence of chiggers. The rodents were captured in Sherman traps (permissions 66/2012, 27/2013 and 41/2013 issued by the Second Local Ethical Commission for Animal Experimentation, Wrocław, Poland), in a deciduous forest in Syców municipal park (51°17'23''N, 17°42'40''E), Poland. The traps were set in a linear or grid-like arrangement, depending on the habitat structure, and baited with dog food pellets. Altogether 5484 larvae were removed from hosts with a dissecting needle and preserved in 96 % alcohol (*n* = 5231) or transferred as live (*n* = 253) to glass vials (24 x 34 mm) filled with charcoaled Plaster-of-Paris and covered with semi-transparent lid. The experimental rearing aimed at ascertaining the relation between the level of engorgement and successful transformation into protonymph.

The degree of larval engorgement was measured for alcohol-preserved and live specimens under stereomicroscope Nikon SMZ800, equipped with ocular micrometer. In calculating the idiosoma volume we used the formula for an ellipsoid, which is almost equivalent to two thirds of a cylinder volume (2/3 (Πr^2^ h)), where r = ½ of idiosoma width and h = idiosoma length). The larvae were assigned to three classes: 1. unengorged specimens or specimens at initial stage of engorgement, with idiosoma volume ranging from 0.0018 to 0.0025 mm^3^, dark orange in colour, with flattened idiosoma and with distinct folds of exocuticle; 2. partly engorged larvae (0.0026–0.0159 mm^3^), with still-retained cuticular folds on non-flattened idiosoma; 3. fully engorged specimens (0.0160 to 0.0228 mm^3^), pale yellow in colour, with smooth, unfolded cuticle. The unequal range of the three classes was due to the observed changes in the shape of the larva’s idiosoma during engorgement, manifest as increase in the length/width ratio. The hypothetical size increase during the parasitic phase was expressed as folds of difference in volume between the largest, fully engorged larva and the smallest, unengorged one.

For the purposes of identification the specimens were mounted on permanent slides in Faure’s liquid. Identification was carried out in compound microscope Nikon Eclipse E600, equipped with differential interference contrast. In ascertaining the specific affiliation of larvae we followed Kudryashova [19] and Stekolnikov [20].

Parasite population parameters, such as prevalence, abundance and intensity of infection, supplemented with data on the highest and lowest infection rates, were calculated. Statistical analysis (Kruskall-Wallis test) aiming at comparison of abundance between the three rodent hosts was performed in Statistica 12 software [21].

## Results

**Fig. 1:**
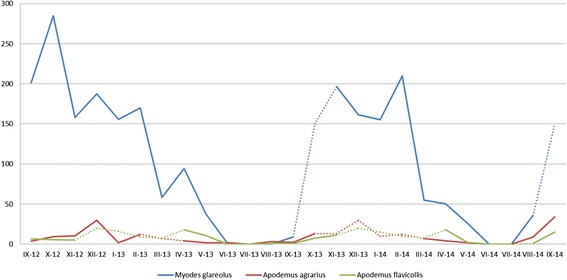
Seasonal dynamics of mean abundance of *H. zachvatkini* on rodents in the study area. Segments corresponding to null returns in rodent catches indicated with dotted line

The collected chiggers represented *Hirsutiella zachvatkini* (Schluger, 1948). The onset of mass appearance of larvae on hosts, inferred both from the pattern of seasonal abundance (Fig. 1) and from the highest proportion of unfed mites recorded on hosts (Table 1), fell on the turn of summer and autumn.

**Table 1:** Proportion (%) (a) and total number of larvae (b) assigned to each engorgement class; – null returns in Sherman traps, *catches of non-infected rodents

		*Myodes glareolus*			*Apodemus agrarius*			*Apodemus flavicollis*		
		1^st^ class	2^nd^ class	3^rd^ class	1^st^ class	2^nd^ class	3^rd^ class	1^st^ class	2^nd^ class	3^rd^ class
IX 2012	a	11	59	30	0	60	40	0	0	100
	b	44	239	120	0	9	6	0	0	13
X 2012	a	0	57	43	0	54	46	9	18	73
	b	0	162	123	0	20	17	1	2	8
XI 2012	a	0	60	40	0	74	26	0	20	80
	b	0	98	64	0	23	8	0	1	4
XII 2012	a	1	64	35	0	60	40	–	–	–
	b	10	666	365	0	71	48	–	–	–
I 2013	a	1	63	36	0	0	100	–	–	–
	b	4	294	169	0	0	2	–	–	–
II 2013	a	0	71	29	0	50	50	–	–	–
	b	0	121	49	0	6	6	–	–	–
III 2013	a	0	65	35	–	–	–	0	27	73
	b	0	76	41	–	–	–	0	4	11
IV 2013	a	0	52	48	–	–	–	–	–	–
	b	0	245	227	–	–	–	–	–	–
V 2013	a	0	55	45	0	0	100	0	0	100
	b	0	21	17	0	0	5	0	0	11
VI 2013	a	0	33	67	0	50	50	*	*	*
	b	0	1	2	0	1	1	*	*	*
VII 2013	a	0	0	100	–	–	–	*	*	*
	b	0	0	1	–	–	–	*	*	*
VIII 2013	a	*	*	*	0	0	100	0	0	100
	b	*	*	*	0	0	6	0	0	2
IX 2013	a	44	56	0	0	22	78	0	0	100
	b	4	5	0	0	2	7	0	0	1
X 2013	a	–	–	–	0	23	77	0	50	50
	b	–	–	–	0	3	10	0	7	7
XI 2013	a	0	19	81	–	–	–	0	5	95
	b	0	108	472	–	–	–	0	1	23
XII 2013	a	0	43	57	–	–	–	–	–	–
	b	0	140	184	–	–	–	–	–	–
I 2014	a	0	33	67	–	–	–	–	–	–
	b	0	102	208	–	–	–	–	–	–
II 2014	a	0	27	73	–	–	–	–	–	–
	b	0	113	307	–	–	–	–	–	–
III 2014	a	0	48	52	–	–	–	–	–	–
	b	0	30	33	–	–	–	–	–	–
IV 2014	a	0	44	56	–	–	–	6	47	47
	b	0	22	28	–	–	–	2	16	16
V 2014	a	0	30	70	0	0	100	0	14	86
	b	0	23	55	0	0	2	0	2	12
VI 2014	a	*	*	*	*	*	*	*	*	*
	b	*	*	*	*	*	*	*	*	*
VII 2014	a	*	*	*	–	–	–	*	*	*
	b	*	*	*	–	–	–	*	*	*
VIII 2014	a	0	53	47	0	67	33	–	–	–
	b	0	19	17	0	6	3	–	–	–
IX 2014	a	–	–	–	0	35	65	0	40	60
	b	–	–	–	0	12	22	0	6	9

The analysis of the degree of engorgement of the parasitic larvae revealed a disproportionately small percentage of unfed individuals collected from the three host species, with a distinct predominance of partially and fully engorged ones (Table 1), assigned to the 2^nd^ and 3^rd^ engorgement classes. In total, 65 larvae were assigned to class 1, whereas 2677 and 2742 - to classes 2 and 3, respectively. For the bank vole, the greatest proportion of hungry larvae, 11 and 44 % , was observed in September 2012 and in September 2013, respectively, ranging from 0 to 1 % in the other months. Partially engorged chiggers, assigned to the 2^nd^ engorgement class (*n* = 2485), were almost as numerous as the engorged ones (3^rd^ engorgement class, *n* = 2482). A slight difference between the specimens assigned to 2^nd^ and 3^rd^ engorgement classes (*n* = 153 vs. *n* = 143), though in the absence of larvae assigned to the 1^st^ class, was observed in the striped field mouse. In the case of yellow-necked mouse, larvae of the 1^st^ class were observed exclusively in October 2012 (9 %) and in April 2014 (6 %), at a distinct predominance of fully engorged chiggers (*n* = 117 in the 3^rd^ class vs. *n* = 39 in the 2^nd^ class and *n* = 3 in the 1^st^ class; Table 1).

In laboratory conditions, the larvae assigned to the 2^nd^ and 3^rd^ classes of engorgement, after detachment underwent further development, irrespective of the season. The hypothetical volume increase during the parasitic phase was 12.6 fold at maximum.

**Fig. 2:**
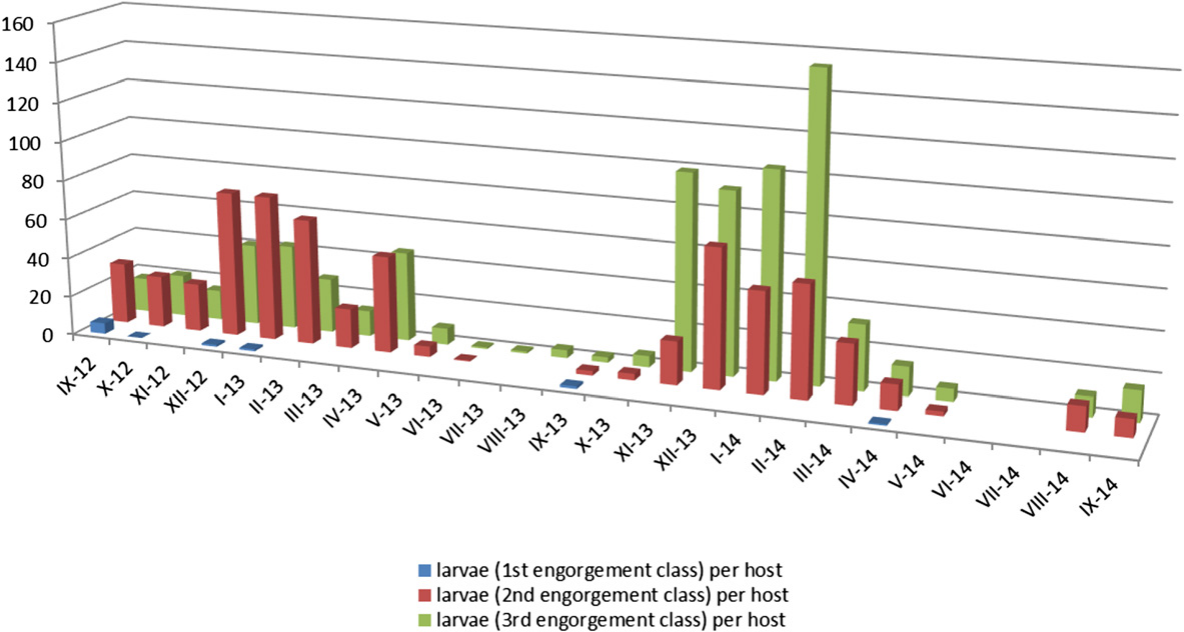
Seasonal dynamics of mean intensity of rodents’ infection with *H. zachvatkini* in the study area

**Fig. 3:**
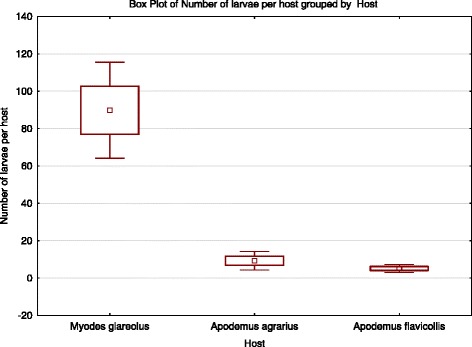
Abundance of *H. zachvatkini* in three rodent species (box – mean +/- standard error, whiskers – 95 % confidence interval)

The highest prevalence (81.3 %) was recorded in *A. agrarius*, with the infection levels ranging from 1 to 69; 77.4 % of *A. flavicollis* were infected with chiggers (infection range: 1–22), whereas the lowest prevalence (75 %) was observed for *M. glareolus*, at the widest range of infection recorded for this host (1–310). The greatest abundance of parasites (including infected and uninfected host individuals) was recorded in the autumn and winter, and considerably decreased during the spring, reaching null values in the early and mid summer (Fig. 1). The mass occurrence on hosts (the highest infection rate) was observed till the late winter (Fig. 2). The differences in abundance among the host species were statistically significant between the bank vole and the *Apodemus* mice (*p* = 0.0002) (Fig. 3). The larvae were attached only to the ears. Their topic preferences depended on the intensity of infection which in turn was season-dependent. In the case of mice, the larvae were found mostly in the external ear canal, whereas the chiggers parasitizing the bank vole preferred the part of the earlobe around the ear hole. When the infection reached its high level in the winter, the chiggers collected from *A. agrarius* and *A. flavicollis* were also attached around the ear hole and those associated with *M. glareolus* – also in the external ear canal.

The strength of attachment to the host, facilitated by the presence of stylostome, varied depending on the degree of engorgement. Among the larvae collected between September and August of the following year, the engorged specimens (2^nd^ and 3^rd^ classes of engorgement) detached easily from the host and continued development into subsequent instars in laboratory conditions, whereas for the smallest ones (1^st^ class of engorgement) induced detachment was not followed by further development.

## Discussion

The seasonal fluctuations in abundance*,* recorded during our study, are similar to those reported by Daniel [22], who noted the appearance of larvae on their hosts in September, their presence during autumn and winter, with a decrease in number in spring, leading to the total absence in summer. The observation corresponds also with laboratory data obtained by Shatrov [5] who pointed to the continuous egg deposition in *Hirsutiella zachvatkini*, with remarkable decrease in July and August. The difference pertains to the absence of larvae on the hosts observed by Daniel [22] in February, which coincided with the finding of unengorged specimens in the upper soil layer. The latter led Daniel [22] to conclude that the larvae, in unfavourable winter conditions, hibernated in the soil before the onset of parasitic phase.

The very small proportion of unfed individuals in the total number of larvae collected from the hosts, at the adopted frequency of catches, indicates a short parasitic phase, initiated soon after establishing the larva-host contact. According to Shatrov [2] the larvae have to start their parasitism relatively soon after getting in contact with the host; otherwise the temperature of the host’s body may lead to their rapid desiccation. The permanent availability of hosts during winter facilitates the early infection. It may also explain the rarity of cases ([22], unpublished observation) of unengorged larvae observed off-host.

The reversed ratio between the 2^nd^ and 3^rd^ engorgement classes from *Myodes glareolus* (and, to lesser extent, from *Apodemus agrarius*) (Table 1, Fig. 2) between November and May of 2012/2013 and the respective months of 2013/2014, should be attributed to different temperature distribution in the two following seasons. The lower temperatures recorded in 2012/2013 compared to 2013/2014 may have had an impact on less intense feeding of larvae, and – in consequence – slower attaining of full engorgement state, contrary to higher temperatures which facilitated the faster feeding which – in turn – resulted in earlier completion of feeding in more favourable thermal conditions.

The percentage of engorged and partly engorged larvae, observed over the survey, supports the hypothesis of developmental arrest in the host-associated instar of *H. zachvatkini*, falling on autumn and winter. After successful attachment, the larvae may maintain contact with the host until they detect environmental signals of favourable conditions for further development. On the other hand, the lack of continuous increase in abundance and intensity towards spring and summer months suggests a gradual detachment of partly and fully engorged larvae, initialized already in autumn, and attaining remarkable values in the spring time. The detachment is likely to be followed by transformation into subsequent instars, as observed under laboratory conditions. It cannot be excluded however that transformation can be arrested at one of the subsequent instars, depending on the environmental conditions.

The ability of various instars of *H. zachvatkini* to hibernate was reported by Daniel [22], Shatrov [5] and Vasil'eva [23]. Much inference was based on laboratory data and field observations are needed to verify the results.

The facultative developmental arrest of larvae is not tantamount to obligatory diapause. The observed seasonality of instars, along with the prolonged duration of larval stage, points to a relatively short development time between the termination of larval instar and the onset of adult instar, i.e. transformation chain, which encompasses the calyptostatic protonymph (PN), predatory deutonymph and calyptostatic tritonymph (TN). The duration of PN and TN, recorded by Shatrov [5], varied between two and four weeks. Such a mode may confirm the non-diapausing nature of calyptostatic instars, as already suggested by Belozerov [18]. It may also explain the relatively rare finds of other developmental instars before the onset of spring, and the absence of records of engorged or partly engorged larvae reported off-host. Another important factor, which may contribute to the developmental arrest of larvae, is the unavailability of food resources exploited by predatory deutonymphs and adults. The correlation between the food intake and successful transformation into subsequent instars was emphasised by Shatrov [5], based on laboratory experiments.

The differences in the level of engorgement of larvae simultaneously associated with the same host should be attributed to time-lagged infection, a phenomenon known also for other parasitengones, including those for which the few-day contact with the host is restricted to food intake. Wohltmann et al. [24] observed larvae of amphibian-associated *Endotrombicula* spp., engorged to a different degree, and the phenomenon was attributed to possible repeated invasions of the hosts by the larvae. A similar observation by Kuo et al. [25] concerned larvae of *Leptotrombidium imphalum* Vercammen-Grandjean et Langston, 1976 associated with three rodent species: *Rattus losea* (Swinhoe, 1871), *Bandicota indica* (Bechstein, 1800) and *Apodemus agrarius.* Also in the course of our study the unengorged larvae were observed on the host (*A. flavicollis*) in the spring (Table 1), thus confirming the possibility of continuous invasions. Much indicates that the time-extended oviposition and asynchronous development of eggs contribute to this phenomenon. According to Shatrov [2], one female of *H. zachvatkini* can continue laying eggs for more than 200 days, whereas the duration of egg and prelarval instars can extend up to 109 days for central-European representatives of the species. The observations were carried out in laboratory, at ambient temperatures varying between 16 and 26 °C [2] or 15 and 25 °C [5], which match the thermal conditions of rodent burrows and overlap with the range 17–19 °C, stated by Daniel [26] for bank vole burrows. Shatrov [5] and Shatrov and Kudryashova [27] pointed also to the prolonged potential of eggs for further development after dormancy of 300–400 days [5] and to the maintained readiness of the larvae to infest their host after relatively long pre-parasitic life (200 days or more [27]). However, the difficulties in obtaining unfed larvae were attributed by Shatrov [5] to the “very long diapauses on the egg stage and to a quite irregular prelarvae development”.

Knowledge of the duration of actual parasitic phase in terrestrial parasitengone larvae, along with the actual time required for transformation into subsequent (protonymph) instar, contributes to verification of the hypothesis on the time-extended, not limited to feeding, association of larvae of *H. zachvatkini* with their hosts. Among the larvae collected in the late summer, autumn and winter season, the engorged specimens (2^nd^ and 3^rd^ engorgement classes) after self-detachment developed into protonymphs in laboratory conditions. A lag of three weeks at most between the subsequent collections carried out on the turn of summer and autumn, at remarkable increase in number of larvae (including partly and fully engorged ones) shows a possibility of completing the parasitic phase in less than 21 days, albeit the actual time may vary to some extent, depending on environmental conditions. Stimuli which cause self-detachment need to be examined in detail, however the physiological response of host to stress caused by trapping, not corresponding with the signals which may be of principal importance at unaffected conditions, seems to constitute one of the important factors facilitating the early detachment. The ease of larval detachment, irrespective of the degree of engorgement, may depend also on the structure of stylostome, and thus may vary among species. According to Shatrov [1] the stylostome of *H. zachvatkini* is relatively short, compared to other trombiculid species. We could observe the differences in the strength of attachment between similarly engorged larvae of *H. zachvatkini* and *Neotrombicula* sp. [unpublished observation], and we hypothesise the presence of longer feeding canal in the latter species.

The remarkable difference in size between the unengorged and the fully engorged larvae, despite the gradual stretching of cuticular folds within the idiosoma, may also be enhanced by neosomy observed in some parasitengone taxa [28–30]. At present, however, there is no evidence for a positive correlation between the neosomy and the prolonged association with the host. Also, the relatively short parasitic phase has been demonstrated for both the neosomatic parasitengones and those for which neosomy has not been confirmed. As observed by Wohltmann [30], the size increase of up to 25-fold is facilitated by stretching of the pre-folded parts of the exocuticle, whereas the mass increase, exceeding the initial volume more than 25-fold, should be attributed to neosomy. In view of the above criterion, there are no reasons to regard neosomy as a phenomenon occurring in *H. zachvatkini*.

As far as parasite population descriptors are concerned, the prevalence of *H. zachvatkini* reported by Miťková et al. [31] in five rodent species was 16.84 %, whereas the intensity varied between 0.13 and 1.84, depending on the host, reaching the highest value in *M. glareolus*. According to Daniel [22], both abundance and intensity are higher in Microtinae than in Murinae.

Kuo et al. [25] observed that the infection rate was positively correlated with the host’s body size, which is incompatible with our observations. For the smallest species, the bank vole, we recorded the highest infection rate, whereas the biggest host, the yellow-necked mouse, carried the smallest load of larvae. However, the overall degree of engorgement observed in the larvae collected from larger hosts was higher compared to those from smaller hosts.

The attachment sites preferred by trombiculid larvae are body parts covered with thin, hairless skin, mainly within head and ears, armpits, abdomen, genitalia and the region of tail and anus. Chiggers parasitizing reptiles and amphibians are reported to reside in special skin folds known as mite pockets [32] or capsules [33]. Benedek et al. [34] reported that larvae of *Neotrombicula autumnalis* parasitising *A. flavicollis* were mostly found in the ears and only 9.8 % were attached to the genitalia. The larvae of *H. zachvatkini* collected from three rodent species during our survey were attached exclusively to the ears, which is compatible with Shatrov’s [1] observations. At the seasonal peak of abundance, however, the larvae expanded the occupied areas beyond the most preferred ones. According to Traub and Wisseman [3] the topic preferences depend on thigmotactic response in chiggers. Due to the latter, the larvae may form clusters on the host body, consisting of mites in the same degree of engorgement which indicates simultaneous attachment, or they attach to a part of the host’s body providing mechanical stimuli dorsally or laterally. Moreover, in their studies on *Leptotrombidium deliense* (Walch, 1922) found on small mammals, Traub and Wisseman [35] noticed that the actual attachment sites were host species-dependent (i.e. tragus of the ear - on rats; belly and inguinal region - on tree shrew; thighs - on soricid shrews), which is consistent with our observations of the attachment sites within the aural area, different in *Apodemus* mice and in the bank vole. Traub and Wisseman [35] explain trombiculid topic preferences by the grooming habits of their hosts.

## Conclusions

The developmental arrest of rodent-associated larvae of *H. zachvatkini* favours intra-population simultaneity of transformation into subsequent instars, which may undergo relatively quick alternation from protonymph to adult. It facilitates the larval survival over winter and synchronization of instars with the availability of food resources exploited by predatory deutonymphs and adults. Due to the obvious dominance of larvae, observed in late summer, autumn, winter and spring, the strategy seems to predominate over alternative modes, in which the developmental delay of deutonymphs and adults can be observed. The developmental delay of larvae should not be identified with diapause, also its identity with hibernation is disputable. Engorged and partly engorged larvae gain the ability to transform into subsequent instars in less than two weeks, however the onset of protonymph instar is most probably withheld in the wild due to some environmental signals and despite the comparable thermal conditions in the wild and in the laboratory. The size increase of larvae during the parasitic phase does not corroborate the neosomy. The topic preferences of larvae become less obvious at maximum infection rates. Quantitative descriptors of parasite population place *M. glareolus* among the most infected hosts of *H. zachvatkini* in contrast to *A. flavicollis* and *A. agrarius* from the same habitat.

The results, though contributing to the life strategies at population level, should gain support from intense studies on environmental signals that may play a role in alternation of instars at natural conditions.
